# The Hospital World

**Published:** 1911-06-24

**Authors:** 


### The Hospital World.

### Resignation of a Dispensing Staff.

In spite of the protests of the medical staff, the management of the Sunderland Dispensary has continued the distribution of a leaflet setting forth the advantages of membership of the dispensary, and last week things were brought to a head by the resignation in a body of the twelve members of the medical staff.

### The Total of the Prince Francis Memorial Fund.

So prominent have been the activities of the promoters of this fund for the Middlesex Hospital that the present grand total realised by the series of entertainments which they have organised becomes of interest. Since November 1 last when the fund was opened £28,361 have been raised, and it is much hoped that £30,000 will be reached by the Coronation.

### A Millionaire and Two Hospitals.

Widespread attention has been drawn to the will of the late Mr. Walter Savill, ship owner and broker, not only because he left over a million and a half, and stated that any individual's share would be forfeited who "shall not profess the Protestant religion," but because of its two bequests to hospitals. The Sussex County Hospital, Brighton, receives £5,000, and the Royal Hospital for Incurables, Putney, £2,000. It has been stated that the duties payable on the estate will amount to about £260,000.

### Doctor Shot in Paris.

The murderous attack on Dr. Guinard, who received four revolver-shots as he was leaving the Hôtel Dieu Hospital, at which he was house-surgeon, may be thus summarised from the *Figaro*. At half-past twelve Dr. Guinard, after a friendly good-bye to his pupils, was walking through the great *cour d'honneur* when a midwife stopped him to speak about one of his patients. All at once an individual confronted him, and, brandishing a Browning revolver of heavy calibre, fired four bullets into him. The would-be murderer, a well-dressed young man, did not attempt to fly. His declaration to the police reads: "My name is Luis-Candedo Herrero. I was born at Barcelona in 1873. I am a tailor, and I live at Colombes (Paris)." Interrogated as to the motives for his attack, he said that he had been operated on by Dr. Guinard for fistula some eleven months ago at the Hôtel Dieu Hospital, adding, "He cut me up as if I had been a pig! and I vowed vengeance from that moment." It is thought that Herrero is out of his mind. Since writing the above we learn that Dr. Guinard has died of his wounds. His confrères, who asked for the rosette of Officer of the Legion of Honour for him, learned that the Prime Minister had recommended him too late to make the news of value to their patient. Dr. Guinard was born in 1858, and was a practical and theoretical exponent of surgery.

### A French Doctor on Algerian Hospitals.

Doctor Henri Fauvel, formerly a practitioner at Havre, and known in France as one of the doctor poets of Normandy, has contributed some notes on a visit to Algeria and Tunis to recent issues of *La Chronique Médicale*. His long experience on the staff of the Havre Hospital enables him to afford concise intelligence touching various medical institutions in the aforesaid countries. He speaks favourably of the Mussulman natives of Algeria as patients. It is curious to hear that from the first days of Algeria's conquest the French doctor was regarded by the majority of the natives as "a sacred personage to whom God has given power to drive away sickness." "Algiers," he proceeds to state, "possesses renowned hospitals: the Civil Hospital of Mustapha, the Military Hospital of the Dey at Bab-el-Oued. The poor are well cared for in the hospitals at Tunis, *i.e.* the new French Civil Hospital, the native Sadiki Hospital, the Italian Hospital, the Jewish Hospital. These last two institutions, however, are too confined: they would do well to be extended and to abandon the old buildings which restrict their limits. The Military Hospital (Hôpital du Belvédère), which is situated in the country on the Ariana route, is injuriously exposed to the south to the sirocco, which often renders it uninhabitable. It is also without gas. The diminution of the troops of Tunis, however, makes these inconveniences less important. Above all others the Sadiki Hospital is the one to visit. All the up-to-date perfections of modern clinics and surgery are to be met with there. Three eminent men have done wonders in its restoration, namely, Si Béchir Sfar, the director of the *Habours*, on whom the hospitals are dependent; Doctor Brunswic-le-Bihan, head surgeon of this hospital, and Dr. Ch. Comte, of the Pasteur Institute, who was the patient organiser of the present surgery, and the creator of the incomparable service of sterilisation. But if the city of Tunis is well provided with hospitals, this is not the case throughout the rest of the Protectorate. In some parts there are very inadequate dispensaries, in others none at all, or next to none. There remains much to be done in this respect."

### An Exceptional Career.

The recent death of Dr. Edward Henry Douty, of Cannes and Stratford-on-Avon, at Cannes, had a peculiar interest to Cambridge men. His practice amongst undergraduates at one time was very extensive, and was fostered by his conversational powers and ready manner. Dr. Douty had a varied and extensive education at St. Edmund's, Salisbury, Vienna, St. Bartholomew's and Middlesex Hospitals, and the Universities of Cambridge, Paris, and Lausanne. He took his M.A. and M.D. of Cambridge in 1898; his M.D. of Paris in 1904; his F.R.C.S. in 1906. Among still other degrees he held the M.D. of Lausanne (1901) and the Federal Diploma of Geneva. His multitudinous appointments had included those of Demonstrator of Anatomy at Cambridge University in 1887, and house surgeon at Middlesex Hospital, lecturer in Cambridge University, member of the Cambridge County Council, surgeon to the Queen Victoria Memorial Hospital at Nice, and to L'Asile Evangélique at Cannes, gynæcological surgeon to Addenbrooke's Hospital, and clinical assistant Royal London Ophthalmic Hospital. Many scientific societies claimed him as a member. His attempts as an author included a publication in German on "Quicksilver in Syphilis," his French thesis on "Le Sanatorium Idéal," and various papers in the Transactions of the Cambridge Medical Society. This bare record is enough to show the energy which Dr. Douty put into his life. He was only fifty years of age. It has since been written of him that "there were few subjects on which he had not clear and independent views. Though he had written comparatively little, his striking personality exerted a direct and widespread influence over his pupils and patients, especially the undergraduates at Cambridge between 1885 and 1897. No one was less like the traditional Don, but . . . he had a shrewd eye both for humbug in high places and for merit in unlikely subjects."

